# Nomograms of risk prediction and prognosis for the T1-T2 stage gastric cancer with lymph node metastasis: a population-based study

**DOI:** 10.3389/fmed.2025.1492041

**Published:** 2025-02-25

**Authors:** Guo-Le Nie, Longlong Geng, Hao Zhang, Shicheng Chu, Hong Jiang

**Affiliations:** ^1^Department of Colorectal Hernia Surgery, Binzhou Medical University Hospital, Binzhou, China; ^2^The First School of Clinical Medicine of Binzhou Medical University, Binzhou, China; ^3^Department of Major Surgery, Binzhou Medical University Hospital, Binzhou, China

**Keywords:** gastric cancer, lymph node metastasis, nomogram, risk factors, overall survival

## Abstract

**Background and aims:**

Lymph node metastasis plays a crucial role in determining the appropriate treatment approach for patients with gastric cancer (GC), particularly those in the T1–T2 stage. Currently available diagnostic strategies for GC with lymph nodes have limited accuracy. The present research aimed to create and validate diagnostic and prognostic nomograms specifically tailored for the T1–T2 stage GC patients with LNM.

**Methods:**

We derived clinicopathological characteristics of patients diagnosed with GC from the Surveillance, Epidemiology, and End Results (SEER) database. We utilized univariate and multivariate logistic analyses to examine the risk factors linked with the occurrence of lymph node metastasis (LNM) in GC patients within the T1–T2 stage. Furthermore, the prognostic factors related to the T1–T2 stage GC patients with LNM were explored by univariate and multivariate cox analyses. Two nomograms were built by the risk factors screened above.

**Results:**

Ultimately, our study included 5,350 patients with T1–T2 stage GC. After identifying age, T stage, tumor size, primary site, grade, and histological type as risk factors for the LNM occurrence, we successfully developed a diagnostic nomogram utilizing these variables. Age, T stage, M stage, tumor size, primary site, grade, radiation, surgery, and chemotherapy were all independent prognostic factors that related to the T1 – T2 GC patients with LNM. The results of the AUC, calibration curve and decision curve analysis (DCA) showed excellent calibration performance and clinical applicability of the two nomograms. The Kaplan–Meier (K-M) curves clearly demonstrated a notable distinction in overall survival between low-risk and high-risk groups, highlighting the prognostic significance of the nomogram.

**Conclusion:**

The establishment and validation of the two nomograms for T1-T2 GC patients with LNM were successful, serving as valuable tools for clinical decision-making and the formulation of personalized treatment approaches.

## 1 Introduction

Gastric cancer (GC) is a prevalent global cancer, ranking fifth in terms of incidence and third in terms of cancer-related mortality. The presence of lymph node metastasis (LNM) is crucial in determining the appropriate treatment approach and evaluating the patient’s prognosis (1, 2). The main treatment for early gastric cancer is endoscopic resection, while non-early operable gastric cancer is treated by D2 lymphadenectomy with gastrectomy. Therefore, early identification of LNM helps clinicians choose the appropriate treatment and improve the long-term prognosis of patients.

The incidence of LNM varies in GC patients with different depths of tumor invasion (3, 4). Additionally, GC patients without LNM had a higher 5-year survival rate, while GC patients with varying numbers of LNM experienced survival rates ranging from 40 to 80% (5). LNM diagnosis of gastric cancer is mainly based on multidetector-row computed tomography (MDCT), but its accuracy rate is only 60–80% (6, 7). There are fewer reports of accurate prediction models for LNM in T1-T2 GC patients. Nomograms have emerged as a powerful tool for integrating and visualizing various clinicopathological variables. They have gained widespread recognition and usage in the diagnosis and prognostication of various diseases (8–10).

In the present study, on the one hand, we explored the risk and prognostic factors based on clinicopathological data of T1-T2 GC patients. On the other hand, we constructed and validated the diagnostic and prognostic nomograms based on the above-screened factors aimed at the identification of LNM status in T1-T2 GC patients.

## 2 Method

### 2.1 Patients selection

We extracted the clinicopathological data of patients with pathological diagnoses of GC in the SEER database between 2010 and 2015. The inclusion criteria: (1) patients who had pathological diagnosis of GC with T1-T2 stage; (2) There were four different histological types of pathology observed, namely adenocarcinoma, squamous cell carcinoma, signet ring cell carcinoma, and carcinoid carcinoma; (3) patients with available clinicopathological data, clear location of the tumor and complete follow-up time. The excluded criteria: (1) patients diagnosed with autopsies or death certificates were excluded; (2) GC patients with survival time < one month; (3) Patients with unclear clinicopathological features were excluded. All GC patients and GC with LNM patients were separated into training and testing groups with a ratio of 7:3. The training group is used for model construction and the testing group is used for model prediction.

### 2.2 Data collection

A total of 12 clinicopathological characteristics, including age, gender, race, T stage, M stage, tumor size, primary site, histological type, surgery, radiation, and chemotherapy were ultimately incorporated into this study. Tumor size is divided into 3 categories: < 20 mm, 20-30 mm, and > 30 mm. It is well known that indolent cell carcinoma is a type of adenocarcinoma. Due to the specific epidemiology and tumorigenesis of signet ring cell carcinoma (SRCC), it is treated as a separate pathological type in this study. Four types of histological types include adenocarcinoma, squamous cell carcinoma, SRCC, and carcinoid carcinoma. The primary site, including cardia, fundus of the stomach (FOS), the body of the stomach (BOS), pylorus, gastric antrum (GA), lesser curvature of the stomach (LCS), and greater curvature of the stomach (GCS). Age, sex, race, T stage, tumor size, primary site, and histological type were used to explore the risk factors related to LNM occurrence. For prognostic factors of LNM occurrence, M stage, surgery, chemotherapy, and radiation were added. The primary outcome measure for the prognostic study component is OS, which is described as the time gap between the date of diagnosis and the date of death from any cause.

### 2.3 Statistical analysis

The statistical analyses for this study were conducted using SPSS 19.0 and R software (version 4.1.2). The Chi-square test was used to compare differences between groups (11). To identify the factors associated with LNM, the researchers conducted univariate logistic regression analysis. The variables that were found to be statistically significant in the univariate analysis were then included in a multivariate logistic regression analysis. This multivariate analysis was used to identify the independent risk factors for the development of LNM in GC patients. Statistically significant variables from the univariate Cox analysis were incorporated into a multivariate Cox analysis.

A *p*-value <0.05 (two sides) was deemed to indicate statistical significance. The caret package is used for dividing the training and validation groups. The receiver operating characteristic (ROC) curves for the nomogram and independent risk factors were plotted by the pROC package (12). Meanwhile, the AUC was used to show discrimination between the diagnostic nomogram and risk factors. Moreover, the calibration curve and DCA were plotted to assess the clinical application value of nomograms (13).

The predictive ability of the prognostic nomogram was evaluated at 12-, 36-and 60-months using receiver operating characteristics (ROC), calibration curves and decision curve analysis (DCA) on the basis of the training group and testing group. The calibration curve analysis was run 1,000 times in repetition via Bootstrap. The calibrate function in the rms package plots the calibration curve. The dcurves package draws DCA. The timeROC package plots time-dependent ROC curves for prognostic normogram in the training and testing group.

Finally, the GC patients with LNM were divided into two groups: low-risk and high-risk, according to the quantile of the risk score estimated using the prognostic nomogram. Statistical differences between the low-risk and high-risk groups were then explored using the K-M curve and log-rank test. The survival and survminer packages are used to compute risk scores and plot K-M curves, respectively (14).

## 3 Results

### 3.1 Baseline characteristics of GC patients

A total of 5,350 GC patients with the T1-T2 stage were finally included. The chi-square test confirms that the allocation between groups is randomized (Table 1). The total population included a total of 1998 (37.35%) cases in males and 3,352 (62.65%) cases in females. Meanwhile, of these patients, 3,665 (68.50%) were T1 stage and 1,685 (31.50%) were T2 stage. 1,497 (27.98%) patients with LNM and 3,853 (72.02%) patients without LNM. The most frequent site of T1-T2 GC is cardia and in 1977 (36.95%) of patients were cardia carcinoma of GC. Adenocarcinoma is the most common pathological type of GC and 4,183 (78.19%) GC patients were pathologically diagnosed with adenocarcinoma.

**Table 1:** Clinicopathologic characteristics between training and validation groups.

Characteristics	Training group (*n* = 3,746)	Validation group (*n* = 1,604)	χ^2^	*p*
Age, years			0.057	0.812
≤65	1,435 (38.3%)	620 (38.65%)		
>65	2,311 (61.7%)	984 (61.35%)		
Gender			0.136	0.713
Female	1,393 (37.2%)	605 (37.72%)		
Male	2,353 (62.8%)	999 (62.28%)		
Race			1.432	0.489
Black	440 (11.7%)	194 (12.09%)		
Other	678 (18.1%)	310 (19.33%)		
White	2,628 (70.2%)	1,100 (68.58%)		
T			0.014	0.907
T1	2,568 (68.6%)	1,097 (68.39%)		
T2	1,178 (31.4%)	507 (31.61%)		
LNM			0.000	0.990
No	2,698 (72%)	1,155 (72.01%)		
Yes	1,048 (28%)	449 (27.99%)		
Primary site			6.200	0.401
BOS	546 (14.6%)	233 (14.53%)		
Cardia	1,383 (36.9%)	594 (37.03%)		
FOS	178 (4.8%)	65 (4.05%)		
GA	899 (24%)	425 (26.5%)		
GCS	215 (5.7%)	81 (5.05%)		
LCS	411 (11%)	163 (10.16%)		
Pylorus	114 (3%)	43 (2.68%)		
Grade			0.004	0.948
I ~ II	1935 (51.7%)	827 (51.56%)		
III ~ IV	1811 (48.3%)	777 (48.44%)		
Histological type			0.083	0.994
Adenocarcinoma	2,926 (78.1%)	1,257 (78.37%)		
Carcinoid tumor	293 (7.8%)	122 (7.61%)		
Signet ring cell carcinoma	503 (13.4%)	215 (13.4%)		
Squamous cell carcinoma	24 (0.6%)	10 (0.62%)		
Tumor size, mm			2.855	0.240
<20	1,405 (37.5%)	601 (37.47%)		
20 ~ 30	958 (25.6%)	442 (27.56%)		
>30	1,383 (36.9%)	561 (34.98%)		

### 3.2 Risk factors for LNM in GC patients

Univariate logistic analysis revealed that age, gender, grade, T stage, histological type, primary site, and tumor size were all found to be significant risk factors. The multivariate logistic analysis results showed that the age, T stage, tumor size, grade, primary site, and histological type were independent risk factors for LNM in GC patients (Table 2).

**Table 2:** The results for univariate and multivariate logistic regression analyses.

Characteristics	Univariate logistic analysis			Multivariate logistic analysis		
	OR	95%CI	*p*	OR	95%CI	*p*
Age, years						
≤65						
>65	0.807	0.698–0.934	0.004	1.086	0.91–1.296	0.359
Gender						
Female						
Male	1.373	1.181–1.597	0.000	1.071	0.895–1.283	0.454
Race						
Black						
Other	0.960	0.733–1.257	0.767			
White	1.045	0.834–1.310	0.702			
T						
T1						
T2	3.089	2.660–3.588	0.000	2.122	1.784–2.524	0.000
Grade						
I ~ II						
III ~ IV	2.446	2.111–2.835	0.000	1.761	1.467–2.113	0.000
Histological type						
Adenocarcinoma						
Carcinoid tumor	0.101	0.056–0.181	0.000	0.334	0.180–0.619	0.000
Signet ring cell carcinoma	1.053	0.857–1.292	0.624	0.759	0.591–0.974	0.030
Squamous cell carcinoma	2.000	0.892–4.481	0.092	1.515	0.614–3.739	0.368
Primary site						
BOS						
Cardia	1.814	1.440–2.284	0.000	1.029	0.775–1.367	0.841
FOS	0.656	0.418–1.030	0.067	0.707	0.422–1.185	0.188
GA	1.131	0.878–1.457	0.339	1.183	0.88–1.590	0.265
GCS	1.237	0.858–1.783	0.254	1.178	0.776–1.790	0.442
LCS	1.466	1.093–1.964	0.011	1.258	0.895–1.768	0.187
Pylorus	1.371	0.869–2.162	0.175	0.988	0.581–1.680	0.965
Radiation						
No						
Yes	5.750	4.879–6.777	0.000	2.045	1.662–2.517	0.000
Surgery						
No						
Yes	0.561	0.479–0.656	0.000	0.918	0.756–1.115	0.390
Chemotherapy						
No						
Yes	7.981	6.804–9.361	0.000	4.2	3.425–5.150	0.000

### 3.3 Diagnostic nomogram for LNM in GC patients

**Figure 1:**
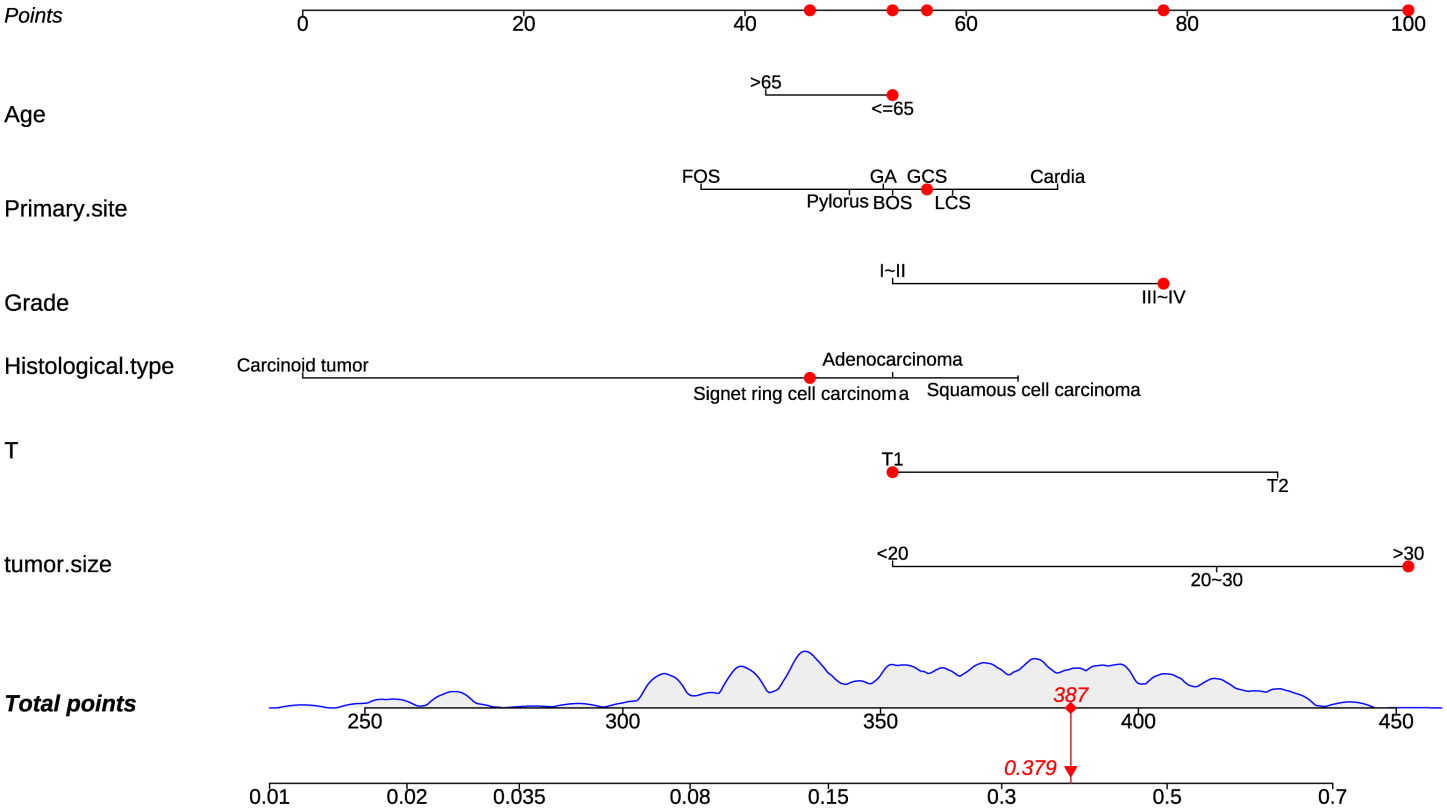
Diagnostic nomogram. Diagnostic nomograms were constructed based on age, T-stage, tumor size, primary site, grading, and histologica type.

**Figure 2:**
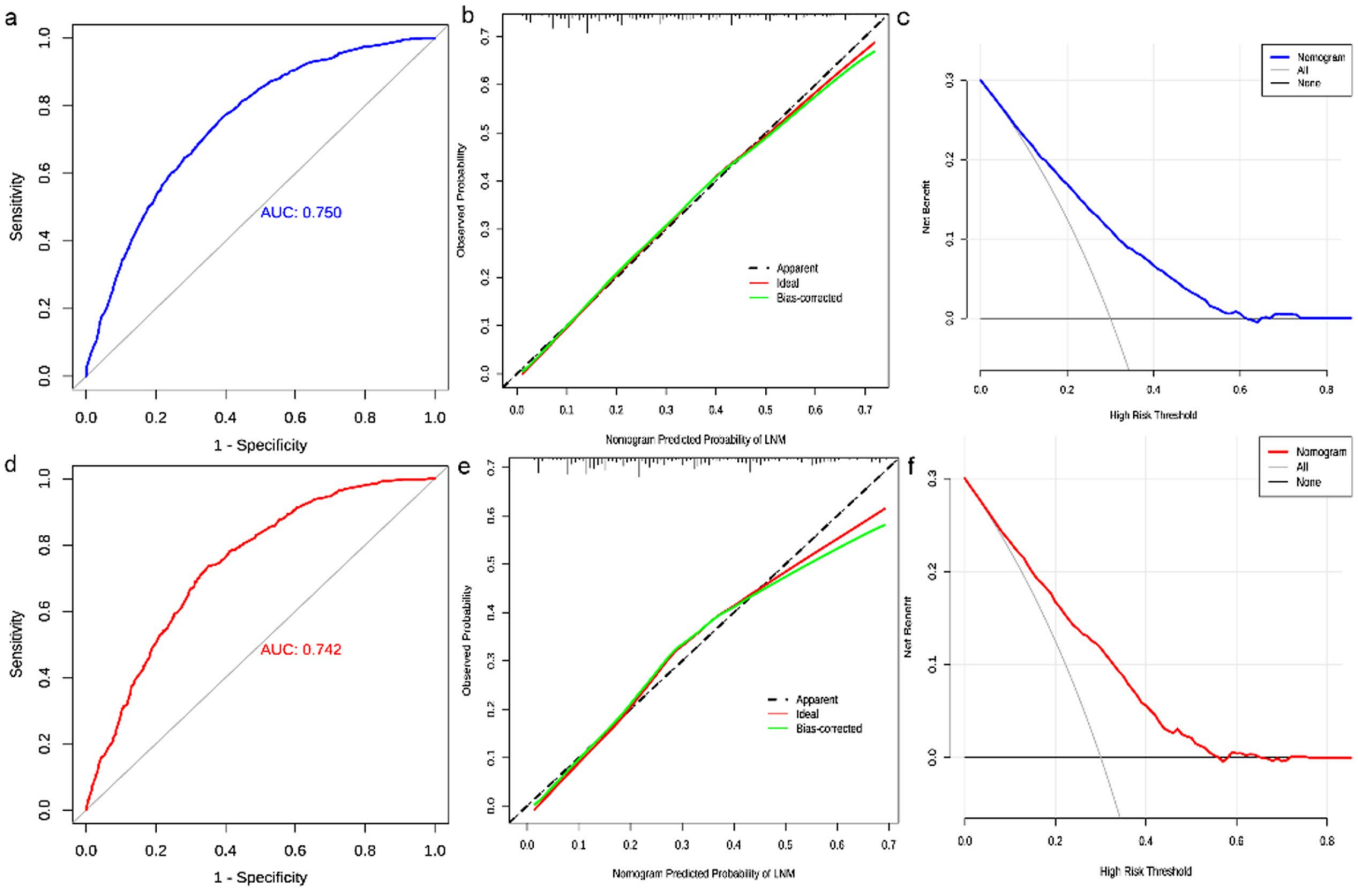
Evaluation of diagnostic nomograms. ROC **(a)**, calibration curves **(b)**, and DCA **(c)** for diagnostic nomograms in the training group. ROC **(d)**, calibration curves **(e)**, and DCA **(f)** for diagnostic nomograms in the testing group.

**Figure 3:**
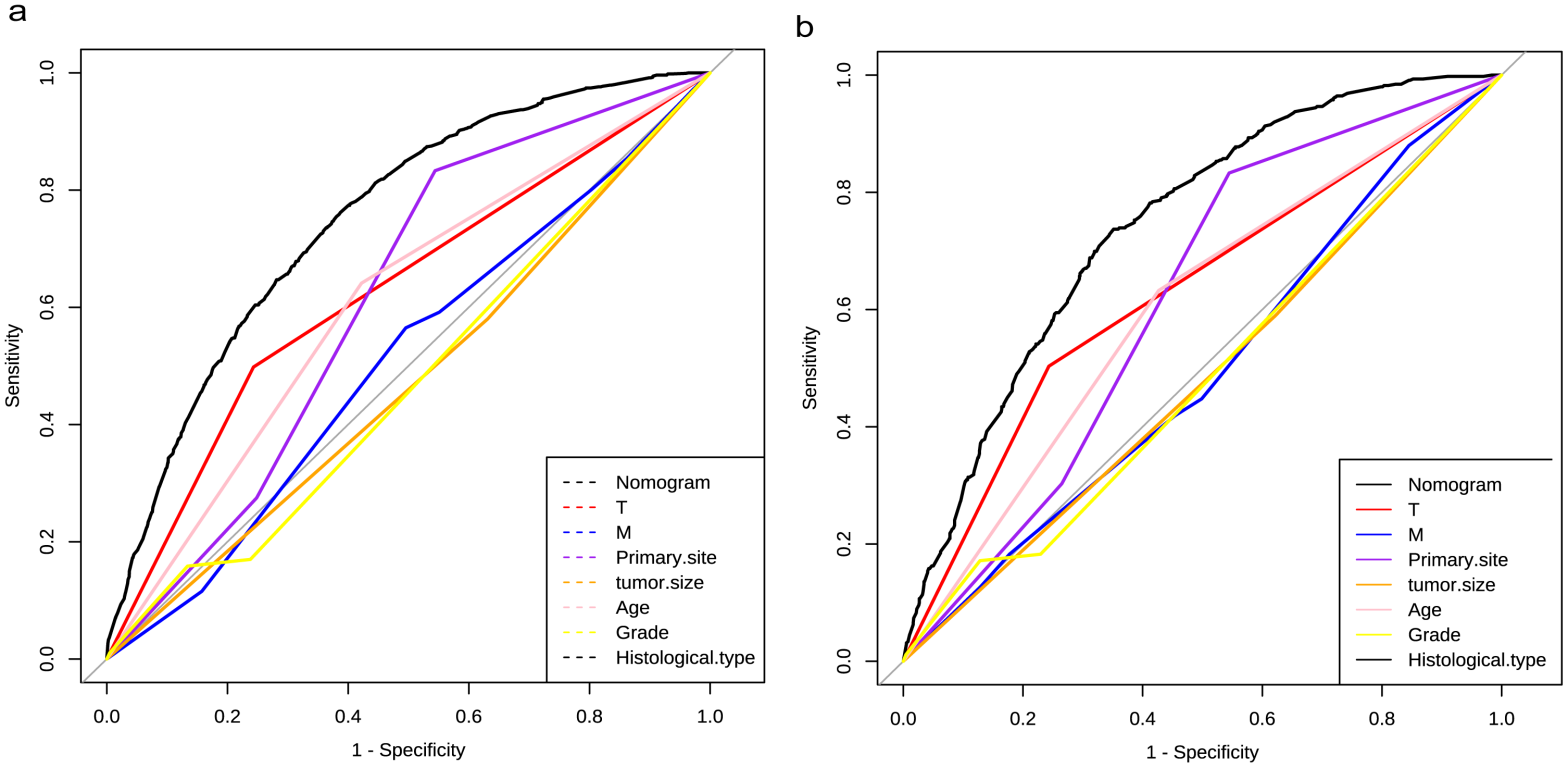
Diagnostic value of diagnostic nomograms. ROCs of nomograms compared with risk factor, including T stage, M stage, primary site, tumor size, age, grade, and histological type, in the training and testing groups.

The diagnostic nomogram was constructed based on the seven independent risk factors (Figure 1). The ROC curves were also plotted and the AUCs were 0.750 in the training group (Figure 2A) and 0.742 in the validation group (Figure 2D). The calibration curves (Figures 2B,E) and DCA (Figures 2C,F) were further plotted and the results showed that the nomogram had an excellent calibrate performance and clinic value. Furthermore, the ROCs of the nomogram and all independent risk factors were plotted and the AUC of the diagnostic nomogram was higher than all independent risk factors (Figure 3).

### 3.4 Characteristics of GC patients with LNM

A total of 1,497 T1-T2 GC patients with LNM, with an incidence of LNM, is about 27.98%. Table 3 summarizes the baseline characteristics of GC patients with LNM. Among these patients, the most common tumor site was cardia and the primary site of 665 (44.42%) GC patients is cardia. Among these patients, 1,015 (67.80%) were male and 482 (32.20%) were female. The histological type for 1,237 (82.63%) GC patients was adenocarcinoma in the present study. There is a random distribution of variables between the two groups, as indicated by the results of the Chi-square test.

**Table 3:** Baseline characteristics for GC patients with LNM.

Characteristics	Training group (*n* = 1,049)	Validation group (*n* = 448)	χ^2^	*p*
Age, years			1.661	0.197
≤65	426 (40.6%)	198 (44.2%)		
>65	623 (59.4%)	250 (55.8%)		
Gender			0.206	0.650
Female	334 (31.8%)	148 (33.04%)		
Male	715 (68.2%)	300 (66.96%)		
Race			0.069	0.966
Black	126 (12%)	55 (12.28%)		
Other	183 (17.4%)	80 (17.86%)		
White	740 (70.5%)	313 (69.87%)		
T			3.201	0.074
T1	509 (48.5%)	240 (53.57%)		
T2	540 (51.5%)	208 (46.43%)		
M			1.410	0.235
M0	792 (75.5%)	351 (78.35%)		
M1	257 (24.5%)	97 (21.65%)		
Primary site			2.409	0.878
BOS	118 (11.2%)	57 (12.72%)		
Cardia	465 (44.3%)	200 (44.64%)		
FOS	29 (2.8%)	12 (2.68%)		
GA	225 (21.4%)	100 (22.32%)		
GCS	57 (5.4%)	24 (5.36%)		
LCS	124 (11.8%)	42 (9.38%)		
Pylorus	31 (3%)	13 (2.9%)		
Grade			0.210	0.647
I ~ II	383 (36.5%)	158 (35.27%)		
III ~ IV	666 (63.5%)	290 (64.73%)		
Histological type			4.838	0.184
Adenocarcinoma	873 (83.2%)	364 (81.25%)		
Carcinoid tumor	8 (0.8%)	9 (2.01%)		
Signet ring cell carcinoma	158 (15.1%)	69 (15.4%)		
Squamous cell carcinoma	10 (1%)	6 (1.34%)		
Tumor size, mm			5.598	0.051
<20	163 (15.5%)	87 (19.42%)		
20 ~ 30	289 (27.6%)	135 (30.13%)		
>30	597 (56.9%)	226 (50.45%)		
Surgery			0.178	0.673
No	356 (33.9%)	147 (32.81%)		
Yes	693 (66.1%)	301 (67.19%)		
Radiation			0.994	0.319
No	561 (53.5%)	227 (50.67%)		
Yes	488 (46.5%)	221 (49.33%)		
Chemotherapy			0.282	0.596
No	318 (30.3%)	142 (31.7%)		
Yes	731 (69.7%)	306 (68.3%)		

### 3.5 Prognostic factors for LNM occurrence

Additionally, the study incorporated three treatment variables, namely surgery, chemotherapy, and radiation, to determine the prognostic factors in GC patients with LNM. The results of univariate revealed that age, gender, race, T stage, M stage, primary site, grade, tumor size, radiation, surgery, and chemotherapy were risk factors for GC patients with LNM. Moreover, the age, T stage, M stage, primary site, grade, tumor size, radiation, surgery, and chemotherapy were independent risk factors identified by multivariate cox analysis. Table 4 summarizes the results of univariate and multivariate cox analyses.

**Table 4:** The univariate and multivariate analyses for GC patients with LNM.

Characteristics	Univariate analysis			Multivariate analysis		
	HR	95%CI	*p*	HR	95%CI	*p*
Age						
≤65	Reference			Reference		
>65	1.370	1.173–1.601	0.000	1.352	1.148–1.591	0.000
Gender						
Female	Reference			Reference		
Male	1.461	1.233–1.73	0.000	1.163	0.973–1.390	0.096
Race						
Black	Reference			Reference		
Other	0.888	0.658–1.198	0.435	0.911	0.672–1.236	0.549
White	1.369	1.076–1.743	0.011	0.933	0.724–1.204	0.595
T						
T1	Reference			Reference		
T2	0.851	0.733–0.989	0.035	1.248	1.061–1.468	0.007
M						
M0	Reference			Reference		
M1	4.201	3.568–4.947	0.000	2.417	1.957–2.985	0.000
Primary site						
BOS	Reference			Reference		
Cardia	2.188	1.661–2.883	0.000	2.116	1.582–2.83	0.000
FOS	1.482	0.892–2.461	0.129	1.258	0.755–2.096	0.379
GA	1.145	0.841–1.559	0.390	1.368	1.002–1.867	0.048
GCS	1.470	0.964–2.242	0.074	1.524	0.995–2.334	0.052
LCS	1.305	0.927–1.838	0.127	1.449	1.026–2.048	0.035
Pylorus	0.469	0.224–0.981	0.044	0.779	0.37–1.641	0.511
Grade						
I ~ II	Reference			Reference		
III ~ IV	1.194	1.021–1.397	0.027	1.409	1.198–1.656	0.000
Histological type						
Adenocarcinoma	Reference					
Carcinoid tumor	0.272	0.068–1.092	0.066			
Signet ring cell carcinoma	0.908	0.735–1.123	0.376			
Squamous cell carcinoma	1.547	0.77–3.108	0.221			
Radiation						
No	Reference			Reference		
Yes	0.582	0.5–0.678	0.000	0.754	0.636–0.893	0.001
Surgery						
No	Reference			Reference		
Yes	0.222	0.19–0.26	0.000	0.372	0.305–0.454	0.000
Chemotherapy						
No	Reference			Reference		
Yes	0.663	0.566–0.776	0.000	0.581	0.486–0.696	0.000
Tumor size, mm						
<20	Reference			Reference		
20 ~ 30	1.528	1.171–1.994	0.002	1.568	1.196–2.056	0.001
>30	2.025	1.588–2.582	0.000	1.694	1.318–2.178	0.000

### 3.6 Prognostic nomogram for GC patients with LNM

**Figure 4:**
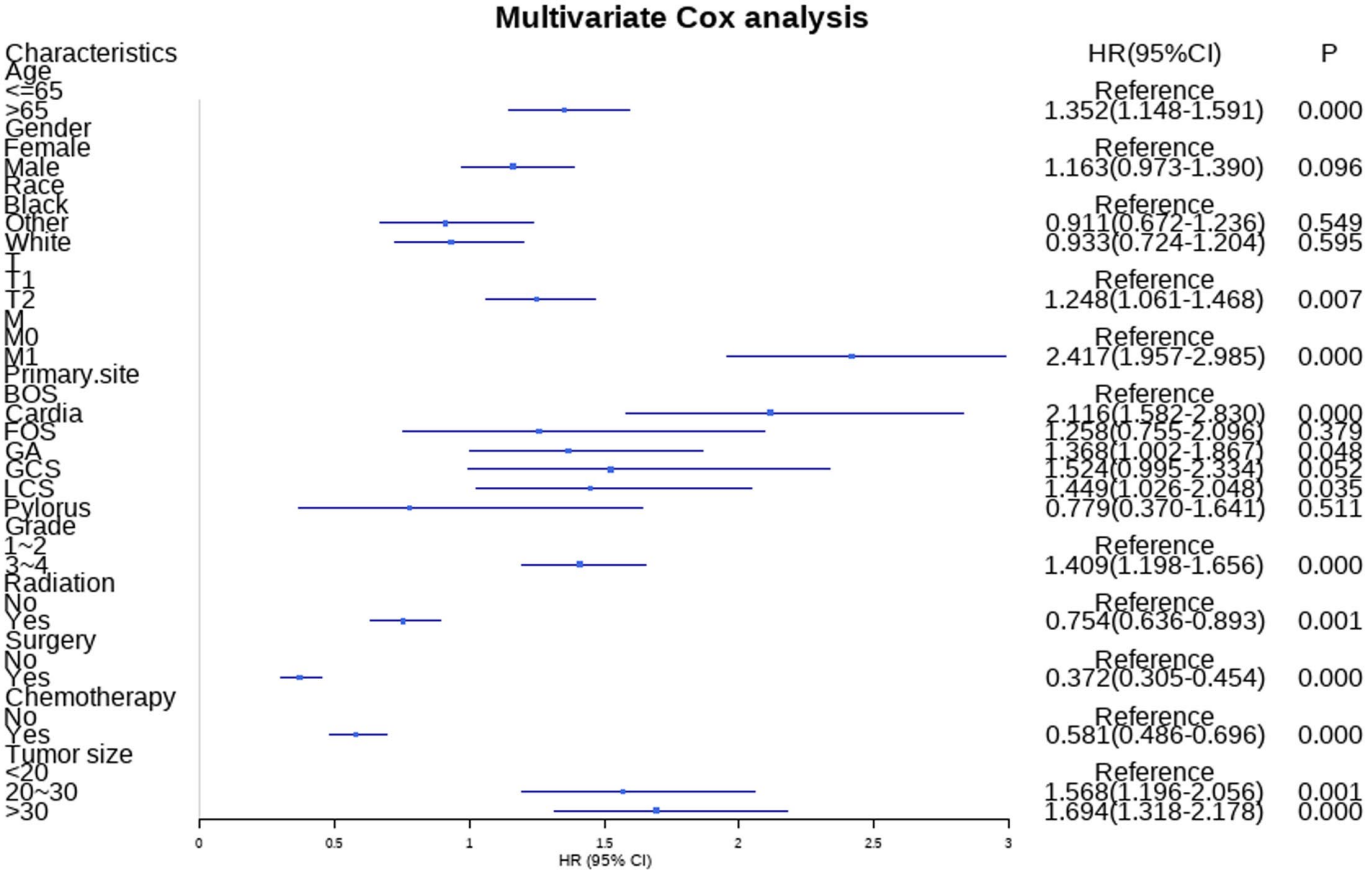
Forest plot of multivariate Cox analysis results.

**Figure 5:**
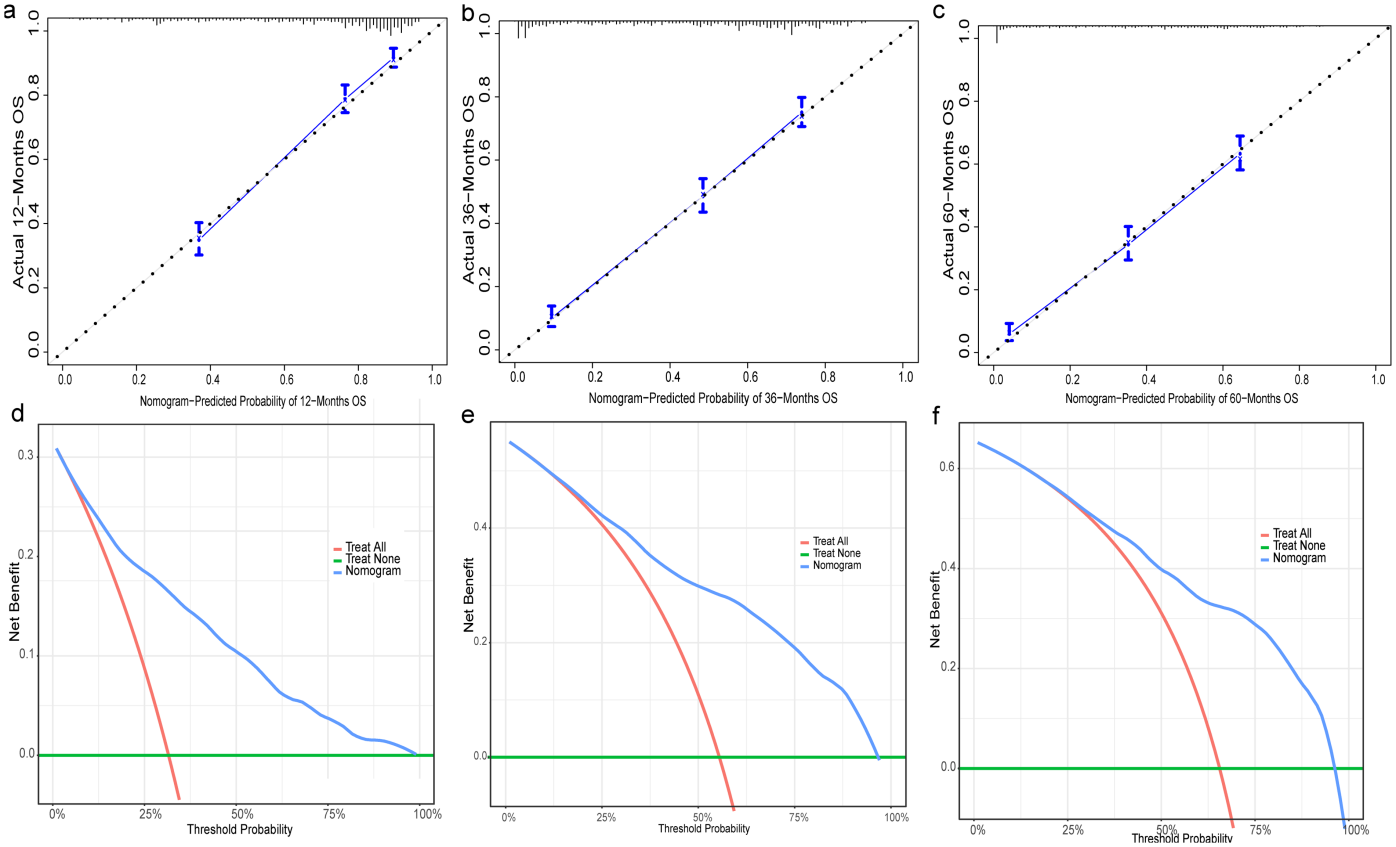
Evaluation of prognostic nomograms in training group. Prognostic nomograms in the training group for 12- **(a)**, 36- **(b)**, and 60- **(c)** month calibration curves and 12- **(d)**, 36- **(e)**, and 60- **(f)** month DCAs.

**Figure 6:**
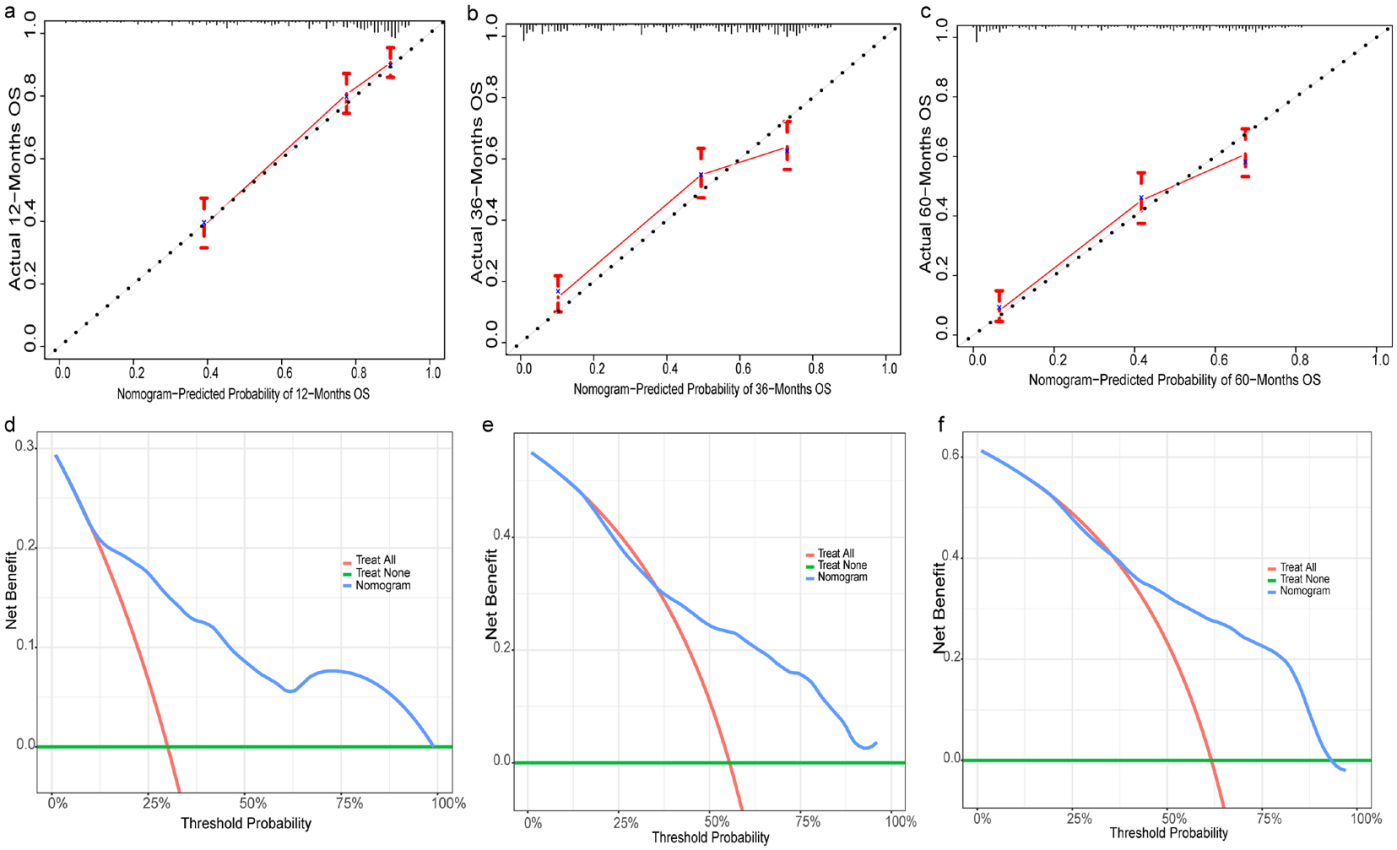
Evaluation of prognostic nomograms in training group. Prognostic nomograms in the testing group for 12- **(a)**, 36- **(b)**, and 60- **(c)** month calibration curves and 12- **(d)**, 36- **(e)**, and 60- **(f)** month DCAs.

**Figure 7:**
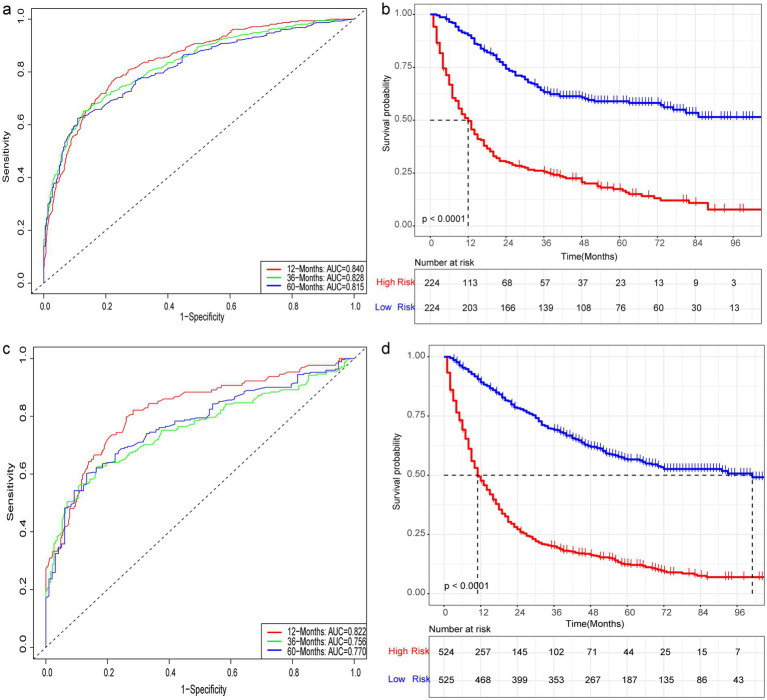
Prognostic evaluation of prognostic nomograms. Prognostic nomograms for 12-, 36-, and 60-month AUC in the training **(a)** and testing **(c)** groups. K-M curves for the high- and low-risk groups in the training **(b)** and testing **(d)** groups after risk stratification based on the prognostic nomogram.

**Figure 8:**
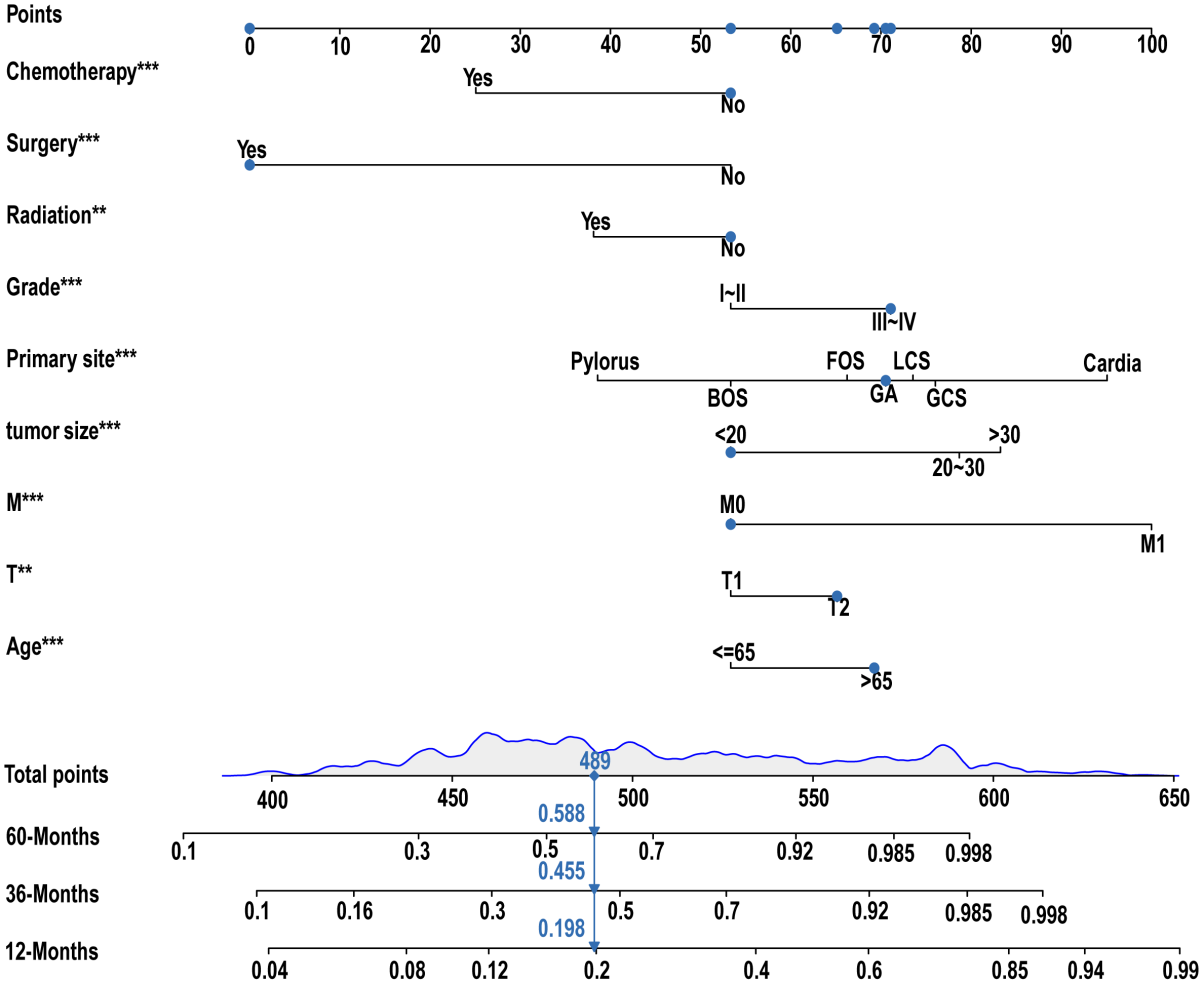
Prognostic nomogram for GC with LNM. A prognostic nomogram for GC with LNM was constructed based on age, T stage, M stage, tumor size, primary site, grading, radiation, surgery, and chemotherapy.

Nine independent risk factors identified by multivariate analysis were used for constructing the prognostic nomogram (Figure 4). Then, the calibration curves and DCA were plotted to validate the prognostic nomograms. The calibration curves both in the training group (Figures 5A–C) and the validation group (Figures 6A–C) showed that the prognostic nomogram had an excellent calibration. Meanwhile, the DCA revealed that the nomogram had a good clinic value both in the training group (Figures 5D–F) and the validation group (Figures 6D–F). Furthermore, the time-dependent ROCs were plotted. The AUCs of 12-, 36-, and 60 months for prognostic nomogram were 0.840, 0.828, and 0.815 in the training group (Figure 7A) and 0.822, 0.756, and 0.770 in the validation group (Figure 7C). Finally, we divided the GC patients with LNM into low-risk and high-risk groups based on the median risk score that predicted by the prognostic nomogram. The KM curves of the two groups showed statistically significant differences in both the training group (Figure 7B) and the validation group (Figure 7D). Prognostic nomogram for GC patients with LNM was shown in Figure 8.

## 4 Discussion

Gastric cancer (GC) is one of the leading causes of cancer deaths worldwide (15). LNM is the most common metastasis site in GC and affecting the long-term survival rate of GC patients. LNM is most often encountered in advanced-stage GC but is less common in EGC. However, the LNM status is critical for treatment strategies for EGC and is commonly associated with poor prognosis. D2 Lymphadenectomy and gastrectomy are among the important surgical procedures for progressive gastric cancer. In addition, in high volume centers treating this lesion, it may be possible to extend lymph node dissection (D2plus) to the para-aorta after exploratory laparoscopic imaging and neoadjuvant therapy studies (16, 17). Treatment decisions for EGC appear to be sophisticated and controversial compared to advanced GC (18). Endoscopic submucosal dissection (ESD) is a treatment modality for early gastric cancer (EGC), but the evaluation of LNM is the most important consideration (19). Therefore, the assessment of LNM status is very important for the treatment and prognosis of patients with stage T1-T2 stage GC.

As medical research techniques continue to advance, more and more studies at the cellular and molecular levels are revealing mechanisms that are closely related to LNM. PRRX1 (20), SOAT1 (21), and MAGEA3 (22) play important roles in the development of LNM in gastric cancer. However, the value of related molecules in clinical applications still needs to be further explored (Figure 8).

Previous studies have reported some clinicopathological factors associated with LNM in GC patients. Milhomem LM et al. retrospectively analyzed 178 patients with EGC and found that ulceration, grade of differentiation, submucosal infiltration, lymphatic infiltration, and vascular invasion were associated with LNM, while the degree of differentiation and submucosal infiltration were independent risk factors (18). A previous study has shown tumor size (> 30 mm), poorly differentiated tumors, and lymphovascular invasion as independent risk factors for LNM (23). In another study, univariate and multivariate analyses showed that age, sex, tumor size, type of differentiation, Lauren’s classification, lymphatic vessel, and perineural infiltration were significantly associated with the incidence of LNM in EGC (24). Meanwhile, the results of a meta-analysis that included 23 studies suggested that gender, age (> 60 years), tumor size (> 20 mm), depth of infiltration, lymphovascular involvement, ulceration, histologic type (non-marked carcinoma), and tumor location (not in the middle of the stomach) were significantly associated with LNM (25). Race was also an independent factor for LNM occurrence (26). In the present study, univariate and multivariate analyses showed that age, T stage, M stage, tumor size, grade, primary site, and histological type were all independent risk factors for LNM, which was similar to the results of the above-mentioned studies. Some differences between the results of different studies still exist, which may be related to the size of the sample and the study design.

Previous studies have also identified many GC-related prognostic factors. Metastasis is one of the prognostic factors of GC (27). Wei et al. showed that age, grade, stage, years of diagnosis, surgery, TNM stage, and tumor size were independent prognostic factors for OS in GC patients with prior cancers (18). Paolo Del Rio et al. revealed that tumor size was an important predictor of survival for GC patients (28). Piotr Kulig et al. showed that T-stage, N-stage, M-stage, and surgery were significantly associated with 5-year survival in GC patients (29). In addition, age is a key factor in the prognosis of GC patients. The 5-year overall survival rate was significantly worse in elderly patients with T1-T1 GC (30). Moreover, the tumor site, T stage, and LNM were significant predictors of disease-free survival for GC in young patients (31).

Up to now, fewer studies have examined the prognostic risk factors associated with GC patients with LNM. In the present study, we found that age, T stage, M stage, tumor size, primary site, and grade were independent prognostic factors for GC patients with LNM. Meanwhile, we also found that surgery, chemotherapy, and radiation were also independent prognostic factors for GC patients with LNM. Early-stage gastric cancer, especially submucosal tumors, has up to 90% survival after surgery (32). In patients with GC extending into the submucosa or with LNM, the results of surgery are poor and usually require chemotherapy, radiotherapy, or chemoradiation to reduce postoperative recurrence rates and improve long-term survival (33, 34).

Most of the current studies on risk factors associated with GC patients with LNM are single-center and small sample size studies. This study has some advantages. The current study included a total of 5,350 GC patients, including 1,497 (27.98%) patients with LNM. At the same time, we constructed predictive and prognostic line graphs with good performance based on easily accessible clinicopathological factors.

Four main case types of gastric cancer were included in our study, namely adenocarcinoma (78.19%), signet ring cell carcinoma (13.42%), carcinoid tumor (7.76%) and squamous cell carcinoma (0.64%). signet ring cell carcinoma is a specific type of adenocarcinoma, and carcinoid and squamous cell carcinomas are very underrepresented. Overall, this paper focused on exploring the correlation between early stage adenocarcinoma patients and the occurrence of lymph node metastases.

There are several limitations to be acknowledged in the current study. Firstly, being a retrospective study, it is prone to selection bias, as the data is collected after the outcome has already occurred. Secondly, the study mainly concentrates on clinicopathological factors, and fails to include important laboratory and imaging measurements that may provide a more comprehensive understanding of the subject matter. Lastly, although the study population sample has undergone external validation, the absence of external data validation from other centers weakens the generalizability of the findings.

## 5 Conclusion

The establishment and validation of the two nomograms for T1-T2 GC patients with LNM were successful, serving as valuable tools for clinical decision-making and the formulation of personalized treatment approaches.

## Data Availability

Publicly available datasets were analyzed in this study. This data can be found at: the datasets for this study can be found in the SEER software (https://seer.cancer.gov/). The research methods involved in the current study can be obtained from the corresponding authors upon reasonable request. No additional information is available.
